# HIV prevention and missed opportunities among people with recently acquired HIV infection: Α protocol for a systematic review

**DOI:** 10.1371/journal.pone.0295462

**Published:** 2024-12-31

**Authors:** Argyro Karakosta, Elisa Ruiz-Burga, Shema Tariq, Giota Touloumi, Emily Jay Nicholls, Nikos Pantazis, Inma Jarrin, Marc Van der Valk, Caroline Sabin, Cristina Mussini, Laurence Meyer, Alain Volny Anne, Christina Carlander, Sophie Grabar, Linda Wittkop, Bruno Spire, Jonh Gill, Kholoud Porter, Fiona Burns, for CASCADE Collaboration

**Affiliations:** 1 Institute for Global Health, University College London, London, United Kingdom; 2 Department of Hygiene, Epidemiology and Medical Statistics, Medical School, National and Kapodistrian University of Athens, Athens, Greece; 3 Central and North West London NHS Foundation Trust, Mortimer Market Centre, London, United Kingdom; 4 National Centre of Epidemiology, Carlos III Health Institute, Madrid, Spain; 5 Centro de Investigación Biomédica en Red Enfermedades Infecciosas, Madrid, Spain; 6 Department of Infectious Diseases, Amsterdam Infection & Immunity Institute, Amsterdam University Medical Centres, Amsterdam, The Netherlands; 7 HIV Monitoring Foundation, Amsterdam, The Netherlands; 8 Department of Surgical, Medical, Dental and Morphological Sciences with Interest Transplant, University of Modena and Reggio Emilia, Modena, Italy; 9 INSERM CESP U1018, APHP Hôpital de Bicêtre, Le Kremlin-Bicêtre, Paris-Saclay University, Gifsur-Yvette, France; 10 Paris, Paris, Île-de-France, France; 11 Department of Infectious Diseases, Karolinska University Hospital, Stockholm, Sweden; 12 INSERM, Institut Pierre Louis d’Epidémiologie et de Santé Publique, Sorbonne Université, AP-HP, Hôpital St Antoine, Paris, France; 13 INSERM, BPH, U1219, CIC-EC 1401, Universite Bordeaux, Bordeaux, France; 14 INRIA SISTM Team, Talence, France; 15 Inserm, IRD, SESSTIM, ISSPAM, Aix-Marseille Université, Marseille, France; 16 Southern Alberta HIV Clinic, Calgary, Alberta, Canada; Southeast University, CHINA

## Abstract

**Background:**

Individuals who have recently acquired HIV represent a unique population because the time frame since HIV acquisition is relatively short and identification of missed HIV prevention opportunities is, therefore, closer to real time and less subject to recall bias. Identifying prevention measures used and missed opportunities for using them, can help stop further HIV transmission.

**Objectives:**

This systematic review aims to synthesise current global evidence on uptake of HIV prevention methods among people with recently acquired HIV from 2007, the year that the concept of ART as a prevention method was first introduced.

**Methods and analysis:**

MEDLINE/PubMed, EMBASE, PsycINFO, Cochrane and Web of Science databases, will be searched for articles published January 2007—December 2023. Eligible studies will be those that reported on HIV prevention methods among people with recently acquired HIV. Quality assessment of the studies selected will be undertaken, and reporting of the systematic review will be informed by Preferred Reporting Items for Systematic Reviews and Meta-Analyses (PRISMA) guidelines.

**Results:**

The systematic review is expected to provide comprehensive insights into the uptake, use and adherence to HIV prevention methods among individuals with recently acquired HIV. Analysis anticipates identifying gaps in prevention coverage, missed opportunities for intervention, and variations in access to and use of prevention strategies. Sociodemographic, personal, and behavioural factors influencing prevention uptake and adherence will also be synthesised.

**Conclusions:**

The findings will be of key relevance to researchers, healthcare providers including third sector organisations/ community groups and policymakers, as they will offer insight into better understanding of missed or failed HIV prevention efforts and will help ensure future efforts meet the needs of those in need of them.

## Introduction

HIV prevention has changed dramatically over the past three decades and effective combination prevention, that includes Pre-Exposure Prophylaxis (PrEP) and treatment as prevention (TasP) [1, 2] as components, means elimination of HIV transmission is now feasible. As a consequence, prevention is a key component of the ambitious agenda set in 2021 by the global HIV community, “End Inequalities. End AIDS”. As part of the United Nations Declaration on Ending AIDS [3], Member States also committed to 95% of people at risk of HIV infection having access to appropriate, person-centred and effective combination prevention options by 2025. However, despite antiretroviral therapy (ART) being freely available to people living with HIV for many years [1] and widening access to PrEP [4, 5], HIV transmission remains high, with around 1.5 million people estimated to have acquired HIV in 2021 [6]. Multiple HIV prevention strategies exist to reduce the risk of HIV acquisition and transmission; however, they remain underutilised [7].

Recently-acquired HIV plays a critical role in ongoing HIV transmission due to the unique characteristics of early infection. Acute and primary HIV infection, which occur within the first six months following acquisition [8], are marked by extremely high levels of viraemia that significantly increase the likelihood of onward transmission [9]. This heightened infectiousness during early infection can disproportionately contribute to the spread of HIV within populations. People with recently acquired HIV are most likely to accurately recall the circumstances around acquisition, allowing identification and understanding of missed opportunities for HIV prevention. Studies of individuals with recently acquired HIV can provide useful insights for HIV prevention efforts and lead to a decline of transmission.

We propose undertaking a systematic review to synthesise current global evidence on access to and uptake of HIV prevention interventions among people with recently acquired HIV. To prevent duplication of reviews, a preliminary search of similar protocols and reviews was conducted in July 2024 using the Cochrane Library, MEDLINE, Embase, PubMed, Web of Science, PsycINFO and The International Prospective Register of Systematic Reviews (PROSPERO) databases. No review protocol or systematic review on this topic was identified. Rather, systematic reviews on recently acquired HIV, identified through searches of these databases, examined the diagnostic, clinical and public health implications of identifying and treating people with recently acquired HIV [10–18]. A consistent theme among these studies was the important contribution of people with recently acquired HIV to HIV epidemics, as several groups have reported disproportionate rates of onward HIV transmission from individuals with acute infection [10–18], although this issue remains controversial [11]. According to these studies, transmission clusters tend to be driven by recently acquired undiagnosed infection, and although estimates are highly variable, recently acquired HIV has been identified as the source for between 10% and 50% of all transmissions [18]. During the earliest stages of HIV infection plasma viral load levels increase exponentially, and this comprises a dominant factor predicting transmission to sexual partners [19]. In addition, discrepancies in the reported contribution of recently acquired HIV infection to ongoing epidemics could be explained by differences in epidemic stage, definitions of “early HIV”, and variation in sexual behaviours (i.e., anal vs. vaginal intercourse, partner change rates, etc.) [13, 18, 19]. Even in a well-established HIV epidemic where the role of recently acquired HIV generally appears to be lower, detection of persons with recently acquired HIV provides an opportunity to intervene at the earliest possible stage of infection [12] to reduce the risk of missed opportunities for prevention of onward HIV transmission.

A better understanding of missed or failed HIV prevention opportunities will help ensure prevention efforts better meet those in need of them. This systematic review aims to synthesise current global evidence on uptake of HIV prevention methods among people with recently acquired HIV. Specifically, this systematic review seeks to answer the following questions: 1) What HIV prevention methods have people with recently acquired HIV used, if any? 2) What are the sociodemographic, personal and behavioural factors associated with uptake, use of, and adherence to HIV prevention methods?

## Materials and methods

This will be a systematic review designed to summarise evidence of access to and uptake of HIV prevention methods among people who had recently acquired HIV infection. The review will commence in December 2023. This protocol has been developed in accordance with the Preferred Reporting Items for Systematic Review and Meta-Analysis Protocols (PRISMA-P) guidelines [20] [S1 Appendix] and reporting of the synthesised findings will also be informed by PRISMA guidelines [21]. Additionally, this protocol is registered in PROSPERO (CRD42023454414). Important amendments to this protocol will be published along with the results of the systematic review.

This review will be undertaken as part of the CASCADE Collaboration, a mixed methods international study investigating the medical and lived experiences of people with recently acquired HIV (https://www.cascadestudy.net/) [22]. Recently acquired HIV will mean HIV acquisition within the 12 months preceding HIV diagnosis. The 12-month timeframe from HIV diagnosis to define recently acquired HIV aligns with the broader objectives and criteria of the CASCADE study [22]. The longer timeframe may include individuals with lower transmission risk compared to those within the six-month period. However, it also provides a more comprehensive view of recency and ensures inclusion of diverse diagnostic and reporting practices.

For each included study we will document the criteria used to establish recency, with the gold standard being the availability of an HIV-negative antibody test within 12 months of the first positive one, or other laboratory evidence of acute HIV infection.

### Searches

We will search peer reviewed articles on the access and use of HIV prevention interventions among people with recent HIV infection, published in English, from 1 January 2007 to 31 December 2024 (2007 being the year that the concept of ART as a prevention method was introduced [23]). From our search, we will identify studies that assessed at least one of: HIV prevention, condom use, uptake of HIV testing, PrEP, post exposure prophylaxis (PEP), harm reduction (i.e., needle exchange, opioid substitution therapy (OSP), etc.), counselling. The search will be performed in the following databases:

- CINAHL Plus
- MEDLINE
- PubMed
- PsycINFO
- Web of Science
- EMBASE

The following search strategy will be adapted for all databases.

Searches will combine key terms relating to HIV/AIDS (HIV, AIDS, human immunodeficiency virus, etc.) with terms related to recently acquired HIV (seroconverter, acute infection, recent infection, recently acquired, etc.) and terms related to preventions methods (condoms, preexposure prophylaxis, HIV testing, counselling, treatment, combination prevention, etc.). See S2 Appendix for full search strategy.

Additionally, the reference lists of the articles selected for full-text review will be hand-searched to identify any additional eligible articles.

### Types of study to be included

Observational studies, including cohort, case control and cross-sectional studies, as well as randomized controlled trials (RCTs), will be included. RCTs where the intervention is some type of prevention will not be considered, except if baseline information about prevention is mentioned. Qualitative studies will be excluded as our group has recently published a systematic review of qualitative literature on recently acquired HIV [8]. Studies that are not peer-reviewed will also be excluded, as will commentaries, reviews, and conference abstracts. Studies employing mixed methods approaches will be included where the quantitative component is presented in sufficient detail. We will not include grey literature or review articles.

### Participants/population

This review will include studies from any country on adults aged 16 years and over who have recently acquired HIV and where use of at least one HIV prevention method is measured.

The review will exclude studies which focus on people who have recently been diagnosed with HIV, but where the time of HIV acquisition is not known, or if no HIV prevention method is mentioned. We will exclude studies in those aged under 16.

### Exposure(s)

Exposure: (i) Factors associated with reduced uptake, use and adherence to HIV prevention methods or reported as barriers by participants; (ii) Factors associated with increased uptake, use and adherence to HIV prevention methods or reported as facilitators/enablers by participants.

Specifically, we will focus on the sociodemographic, personal and behavioural factors associated with uptake, use and adherence of HIV prevention measures.

### Comparison group(s)

#### Main outcome(s)

The expected outcomes are identifying the prevention methods used, and not used, by people with recently acquired HIV and identifying the barriers to uptake of, use of, and adherence to strategies to prevent HIV acquisition. The outcome of this review is the prior and/or current use of HIV prevention methods among people with recently acquired HIV. This will include mapping of current HIV prevention methods among people with recently acquired HIV.

### Eligibility criteria

This review will include studies which report on adults aged 16 years and over who have recently acquired HIV and where use of at least one HIV prevention method is measured, published between 1/1/2007 and 31/12/2024, and written in English.

### Exclusion criteria

No known time of HIV acquisition/ seroconversion, published earlier than 2007 or recruitment of population earlier than 2007. Studies combining adults and paediatric/adolescent patients will be included only if able to disaggregate results by age to identify those aged ≥16.

### Study selection

Potential articles will be collected in the systematic review software Covidence [24]. Duplicates will be removed by AK using Covidence software. Based on the pre-specified eligibility criteria, studies will be selected using a two-staged approach; titles and abstracts will be examined first and then the full-text of all potential eligible studies will be retrieved and screened. AK and ER-B will independently screen a sample of 100 citations to pre-test and refine coding guidance based on the inclusion criteria and until at least 90% agreement is achieved. Disagreements about eligibility will be resolved through discussion. AK will then screen the remaining citations for inclusion in the review using the pre-tested coding guidance. Full-text of all potentially eligible studies will be retrieved and AK and ER-B will assess all full text articles to determine final study selection. Differences will be resolved through consensus, with input on eligibility from a third reviewer (FB) when necessary. Citations that do not meet the inclusion criteria will be excluded and the reason for exclusion will be recorded at the full-text screening.

### Assessment of risk of bias and quality of evidence

The quality of each included study will be assessed by using the Newcastle Ottawa Scale (NOS) for cohort studies and modified NOS for cross-sectional and case-control studies [25]. We assume that there will be no or very few RCTs on this topic and we will, therefore, use the National Institute for Health and Care Excellence (UK) Quality Appraisal of Intervention Studies tool (derived from Jackson et al., 2006 [26]) as a risk of bias assessment tool. Each paper included in the review will be quality assessed by AK and ER-B. All disagreements will be resolved through consensus. FB will be consulted to discuss any discrepancies in study quality assessment.

### Data extraction

Data will be extracted using a standardised form in Covidence. This will include: 1) characteristics of the study: author, year of publication, study objective, country and context, 2) methodological characteristics: study design, research questions and/or hypotheses, study population, definition of recently acquired HIV used, sample characteristics, type of prevention’/s’ measure/s

If key socio demographic data such as mean age or proportion male is missing, these will be calculated from the data given within the study when possible. Number of participants utilising and not utilising HIV prevention methods in groups exposed and not exposed to each variable of interest (where not stated explicitly, numbers will be calculated from percentages reported in tables), percentage who used HIV prevention and effect measures (odds ratio/hazard ratio with 95% confidence intervals and/or p-values) for variables assessed for association with prevention use.

### Data synthesis

Α narrative (descriptive) synthesis of the data will be conducted, after a final list of papers has been compiled. This discussion will be tailored around the type of prevention strategy, target population characteristics, barriers to uptake, use of and adherence to prevention strategies.

Provided the studies are sufficiently homogeneous and comparable regarding study design, percentages will be pooled through meta-analysis or even meta-regression to explain part of the variability.

Reporting of the systematic review will be informed by Preferred Reporting Items for Systematic Reviews and Meta-Analyses (PRISMA) guidelines [21], as mentioned before.

**PROSPERO registration number.** CRD42023454414.

### Patient and Public Involvement (PPI)

Community members have been involved in the conception and design of this protocol and will contribute to interpretation and dissemination of findings. The involvement of people living with HIV is central to the CASCADE study and takes place at several points during the overall project. Community representatives are involved as members of the CASCADE Executive Committee and the social-science subcommittee, attending steering group meetings, reviewing documents, and contributing to interpretation of findings. The undertaking of this systematic review is supported by community members and they participate as co-authors. More about the role of patient and public involvement (PPI) in CASCADE study can be found elsewhere [22].

### Ethics and dissemination

Ethics approval is not required for a systematic review. Findings of the systematic review will be disseminated through open access publication in a peer-reviewed journal. The findings will be of interest to researchers, healthcare practitioners, policymakers, and HIV community partners.

## Results

We expect that the review will be completed by the end of February 2025. The review will synthesise global evidence on the use of HIV prevention strategies and missed opportunities among individuals with recently acquired HIV infection. Following a comprehensive search and selection process, a narrative synthesis of the identified studies will be conducted, tailored around key themes such as the type of prevention strategy, target population characteristics, and barriers to uptake, use, and adherence to prevention measures. Where sufficient homogeneity exists across studies in terms of design, populations, and outcomes, data will be pooled through meta-analysis. Meta-regression will be employed to explain variability and identify predictors of missed prevention opportunities.

The review will report on the following key outcomes:

1. **Identification of Prevention Gaps:** Highlighting missed opportunities, such as the underutilisation of PrEP, condoms, or other combination prevention strategies, and identifying barriers to access and adherence to these measures.
2. **Patterns of Prevention Uptake:** Providing insights into commonly prior and/or current used prevention methods among people with recently acquired HIV and the sociodemographic, behavioral, and contextual factors influencing their uptake or not.

When quantitative synthesis is not feasible due to insufficient studies or high heterogeneity, a descriptive synthesis will provide an in-depth understanding of prevention-related behaviors and gaps. Subgroup analyses will be conducted where possible to explore factors associated with missed prevention opportunities in specific subpopulations, including: a) key populations, such as men who have sex with men (MSM), people who inject drugs, and sex workers; b) gender differences; c) age groups; d) types of prevention measures; d) country contexts, including developing and developed settings.

## Discussion

Despite new and effective interventions and declines in HIV incidence, a significant number of people continue to acquire HIV. In this systematic review, we are committed to producing evidence that addresses barriers in access to HIV prevention. People who have recently acquired HIV are a unique, and ideal group, in whom to study these clinically and public health relevant questions, and to assess prevention methods used. We believe that our data are extremely relevant to ongoing HIV prevention efforts.

The review will provide evidence to inform the provision of HIV treatment and prevention services. The expected outcomes are identifying the prevention methods used, whether they are used appropriately and effectively by people with recently acquired HIV, and examining the structural and behavioural barriers to uptake, use of, and adherence to HIV prevention measures.

## Supporting information

S1 Appendix PRISMA-P (preferred reporting items for systematic review and meta-analysis protocols) 2015 checklist: Recommended items to address in a systematic review protocol. (PDF)

S2 Appendix Full systematic review database search string. (PDF)

## Abbreviations

HIV: Human Immunodeficiency Virus
ART: Antiretroviral Therapy
PrEP: Pre-Exposure Prophylaxis
TasP: Treatment as Prevention
AHI: Acute HIV Infection
PHI: Primary HIV Infection
PRISMA-P: Preferred Reporting Items for Systematic Review and Meta-Analysis Protocols
PRISMA: Preferred Reporting Items for Systematic Review and Meta-Analysis
PEP: Post Exposure Prophylaxis
OSP: Opioid Substitution Therapy
RCTs: Randomized controlled trials
NOS: Newcastle Ottawa Sca
