# Supplementary material for: HIV prevention and missed opportunities among people with recently acquired HIV infection: Α protocol for a systematic review
Source: PLoS One. 2024 Dec 31;19(12):e0295462. doi: 10.1371/journal.pone.0295462 (PMC11687745; doi:10.1371/journal.pone.0295462)
Supplement: S2 Appendix — (PDF) [file pone.0295462.s002.pdf]

S2 Appendix 2. Full systematic review database search string

Table A. Search strings for Ovid Medline (in-process & other non-indexed citations and Ovid MEDLINE)

#	Search string
1	HIV infections/ or HIV seropositivity/
2	HIV.ab,kw,ti.
3	"Human Immunodeficiency Virus".ab,kw,ti.
4	1 or 2 or 3
5	((recent* or new*) adj3 (infect* or acqui*)).ab,kw,ti.
6	(seroconver* or sero-conver* or recent* HIV infect* or acute infect* or acute HIV or primary HIV).ab,kw,ti.
7	5 or 6
8	(prevent* or PREP or pre-exposure or preexposure or PEP or post exposure or condom* or harm reduction or risk reduction or needle* exchange or opioid subsitut* or syringe or HIV test* or behaviour or behavior or counselling or counseling or HIV transmission or missed opportunit* or treatment or antiretroviral* or ART or sex* or comb* prevent* or TasP).ab,kw,ti.
9	4 and 7 and 8
10	limit 9 to (yr="2007 -Current" and "humans only (removes records about animals)")

Table B. Search strings for Embase

#	Search string
1	Human immunodeficiency virus/
2	HIV.ab,kw,ti.
3	1 or 2
4	((recent* or new*) adj3 (infect* or acqui*)).ab,kw,ti.
5	(seroconver* or sero-conver* or recent* HIV infect* or acute infect* or acute HIV or primary HIV).ab,kw,ti.
6	4 or 5
7	(prevent* or PREP or pre-exposure or preexposure or PEP or post exposure or condom* or harm reduction or risk reduction or needle* exchange or opioid subsitut* or syringe or HIV test* or behaviour or behavior or counselling or counseling or HIV transmission or missed opportunit* or treatment or antiretroviral* or ART or sex* or comb* prevent* or serosort*).ab,kw,ti.
8	3 and 6 and 7
9	limit 8 to (yr="2007 -Current" and "humans only (removes records about animals)")

Table C. Search strings for PubMed

#	Search string
1	HIV[MeSH Terms]
2	HIV seropositivity[MeSH Terms]
3	human immunodeficiency virus[Title/Abstract]
4	HIV infections[MeSH Terms]
5	(#1 OR #2 OR #3 OR #4)
6	recent* infect*[Title/Abstract] OR recent* acqui*[Title/Abstract] OR new* infect*[Title/Abstract] OR new* acqui*[Title/Abstract] OR acute infect*[Title/Abstract] OR

	seroconver*[Title/Abstract] OR sero-conver*[Title/Abstract] OR recent* HIV infect*[Title/Abstract] OR acute HIV[Title/Abstract] OR primary HIV[Title/Abstract]
**7**	prevent*[Title/Abstract] OR PREP[Title/Abstract] OR pre-exposure[Title/Abstract] OR preexposure[Title/Abstract] OR PEP[Title/Abstract] OR post exposure[Title/Abstract] OR condom*[Title/Abstract] OR harm reduction[Title/Abstract] OR risk reduction[Title/Abstract] OR needle* exchange[Title/Abstract] OR opioid subsitut*[Title/Abstract] OR syringe[Title/Abstract] OR HIV test*[Title/Abstract] OR behaviour[Title/Abstract] OR behavior[Title/Abstract] OR counselling[Title/Abstract] OR counseling[Title/Abstract] OR HIV transmission[Title/Abstract] OR missed opportunit*[Title/Abstract] OR treatment[Title/Abstract] OR antiretroviral*[Title/Abstract] OR ART[Title/Abstract] OR sex*[Title/Abstract] OR comb* prevent*[Title/Abstract] OR TasP[Title/Abstract]
**8**	(#5 AND # 6 AND #7)
**9**	Humans, Publication date from 2007/1/1

Table D. Search strings for Web of Science

#	Search string
**1**	TS=("HIV" or "HIV infect*" or "HIV seropositiv*" or "human immunodeficiency virus")<br>Indexes=SCI-EXPANDED, SSCI, A&HCI, CPCI-S, CPCI-SSH, BKCI-S, ESCI, CCR-EXPANDED, IC<br>Timespan: 2007-01-01 to 2023-07-24
**2**	TS=((recent* or new*) near/3 (infect* or acqui*))<br>Indexes=SCI-EXPANDED, SSCI, A&HCI, CPCI-S, CPCI-SSH, BKCI-S, ESCI, CCR-EXPANDED, IC<br>Timespan: 2007-01-01 to 2023-07-24
**3**	TS=("seroconver*" or "sero-conver*" or "recent* HIV infect*" or “acute infect*” or "acute HIV" or "primary HIV")<br>Indexes=SCI-EXPANDED, SSCI, A&HCI, CPCI-S, CPCI-SSH, BKCI-S, ESCI, CCR-EXPANDED, IC<br>Timespan: 2007-01-01 to 2023-07-24
**4**	#2 OR #3<br>Indexes=SCI-EXPANDED, SSCI, A&HCI, CPCI-S, CPCI-SSH, BKCI-S, ESCI, CCR-EXPANDED, IC<br>Timespan: 2007-01-01 to 2023-07-2
**5**	TS=("prevent*" or "PREP" or "pre-exposure" or "preexposure" or "PEP" or "post exposure" or "condom" or "harm reduction" or "risk reduction" or "needle* exchange" or "opioid substitut*" or "syrigne" or "HIV test*" or "behavio$r" or counsel$ng" or "HIV transmission" or "missed opportunit*" or "treatment" or "antiretroviral* or "ART" or "sex*" or "comb* prevent*" or "TasP")<br>Indexes=SCI-EXPANDED, SSCI, A&HCI, CPCI-S, CPCI-SSH, BKCI-S, ESCI, CCR-EXPANDED, IC<br>Timespan: 2007-01-01 to 2023-07-24
**6**	#1 AND #4 AND #5<br>Indexes=SCI-EXPANDED, SSCI, A&HCI, CPCI-S, CPCI-SSH, BKCI-S, ESCI, CCR-EXPANDED, IC<br>Timespan: 2007-01-01 to 2023-07-24

Table E. Search strings for the Cochrane Library

#	Search string
**1**	MeSH descriptor: [HIV] explode all trees
**2**	MeSH descriptor: [HIV Seropositivity] explode all trees
**3**	"HIV" OR HIV NEXT infect* OR HIV NEXT seporosit* OR "human immunodeficiency" (Word variations have been searched)
**4**	MeSH descriptor: [Seroconversion] explode all trees

**5**	(recent* NEXT infect* OR recent* AND acqui* OR new* AND infect* OR new* NEXT acqui* OR acute NEXT infect* OR seroconver* OR sero-conver* OR recent* NEXT HIV NEXT infect* OR "acute HIV" OR "primary HIV"):ti,ab,kw (Word variations have been searched)
**6**	MeSH descriptor: [Pre-Exposure Prophylaxis] explode all trees
**7**	MeSH descriptor: [Post-Exposure Prophylaxis] explode all trees
**8**	MeSH descriptor: [Condoms] explode all trees
**9**	MeSH descriptor: [Harm Reduction] explode all trees
**10**	MeSH descriptor: [Risk Reduction Behavior] explode all trees
**11**	MeSH descriptor: [Needle-Exchange Programs] explode all trees
**12**	MeSH descriptor: [Opiate Substitution Treatment] explode all trees
**13**	MeSH descriptor: [HIV Testing] explode all trees
**14**	MeSH descriptor: [Anti-Retroviral Agents] explode all trees
**15**	(prevent* OR PREP OR pre-exposure OR preexposure OR PEP OR "post exposure" OR condom* OR "harm reduction" OR "risk reduction" OR needle* NEXT exchange OR opioid NEXT subsitut* OR syringe OR HIV NEXT test* OR behaviour OR behavior OR counselling OR counseling OR "HIV transmission" OR missed NEXT opportunit* OR treatment OR antiretroviral* OR ART OR sex* OR comb* NEXT prevent* OR TasP):ti,ab,kw
**16**	#1 OR #2 OR #3
**17**	#4 OR #5
**18**	#6 OR #7 OR #8 OR #9 OR #10 OR #11 OR #12 OR #13 OR #14 OR #15
**19**	#17 AND #18 AND #19 (with Cochrane Library publication date from Jan 2007 to Aug 2023, in Cochrane Reviews, Cochrane Protocols, Trials, Clinical Answers, Editorials and Special Collections (Word variations have been searched)

Table F. Search strings for PsycINFO

**#**	**Search string**
**1**	exp HIV/
**2**	((recent* or new*) adj3 (infect* or acqui*)).ab,ti.
**3**	(seroconver* or sero-conver* or recent* HIV infect* or acute infect* or acute HIV or primary HIV).ab,ti.
**4**	2 or 3
**5**	(prevent* or PREP or pre-exposure or preexposure or PEP or post exposure or condom* or harm reduction or risk reduction or needle* exchange or opioid subsitut* or syringe or HIV test* or behaviour or behavior or counselling or counseling or HIV transmission or missed opportunit* or treatment or antiretroviral* or ART or sex* or comb* prevent* or TasP).ab,ti.
**6**	1 and 4 and 5
**7**	limit 6 to yr="2007 -Current"
